# RNA-Seq Perspectives to Improve Clinical Diagnosis

**DOI:** 10.3389/fgene.2019.01152

**Published:** 2019-11-12

**Authors:** Guillermo Marco-Puche, Sergio Lois, Javier Benítez, Juan Carlos Trivino

**Affiliations:** ^1^Bioinformatics Group, Sistemas Genómicos, Paterna, Spain; ^2^Human Genetics Group, Spanish National Cancer Research Center, Madrid, Spain

**Keywords:** RNA-Seq - RNA sequencing, transcriptomics, bioinformatics, clinical routine, tissue-specific expression, variants of uncertain significance (VUS), alternative splicing (AS), DEG (differentially expressed genes)

## Abstract

In recent years, high-throughput next-generation sequencing technology has allowed a rapid increase in diagnostic capacity and precision through different bioinformatics processing algorithms, tools, and pipelines. The identification, annotation, and classification of sequence variants within different target regions are now considered a gold standard in clinical genetic diagnosis. However, this procedure lacks the ability to link regulatory events such as differential splicing to diseases. RNA-seq is necessary in clinical routine in order to interpret and detect among others splicing events and splicing variants, as it would increase the diagnostic rate by up to 10–35%. The transcriptome has a very dynamic nature, varying according to tissue type, cellular conditions, and environmental factors that may affect regulatory events such as splicing and the expression of genes or their isoforms. RNA-seq offers a robust technical analysis of this complexity, but it requires a profound knowledge of computational/statistical tools that may need to be adjusted depending on the disease under study. In this article we will cover RNA-seq analyses best practices applied to clinical routine, bioinformatics procedures, and present challenges of this approach.

## Introduction

In recent years, the use of next-generation sequencing (NGS) for the diagnosis of Mendelian or rare genetic disorders has entered routine clinical practice. The increasing ability to sequence entire genomes in a cost-effective manner has allowed the identification of approximately 260 novel rare genetic diseases per year (Boycott et al., 2017). Focusing on the ∼1.5% of the human genome represented by coding sequences, diagnostic rates of whole-exome sequencing (WES) vary widely by inherited condition, and they range from 28 to 55% (Retterer et al., 2016). By extending the focus to deep intronic and regulatory variants in non-coding regions, including structural and non-exonic variants not detectable by WES, whole-genome sequencing (WGS) increased the diagnostic rate by more than 17% (Lionel et al., 2018). The high rate of undiagnosed cases is related to at least two important limitations: (i) the catalog of Mendelian phenotypes is as yet far from complete (∼300 new Mendelian phenotypes are added to the OMIM database each year (Chong et al., 2015)); and (ii) although the interpretation of protein-coding regions of the genome is reliable, our understanding of non-coding variation and its functional interpretation is still limited.

Recently, different studies reported on how the application of RNA sequencing (RNA-seq) can help to shed light on the possible pathogenicity of variants of unknown significance (VUS) identified through DNA sequencing studies such as WES and WGS, as it provides direct insight into the transcriptional alterations caused by VUS and thus improves diagnostic rates (Cummings et al., 2017; Kremer et al., 2017). Alternative splicing (AS) is considered to be a key cellular process in ensuring functional complexity in higher eukaryotes (Chen et al., 2012). Remarkably, this process is estimated to affect more than 88% of human protein-coding genes (Kampa et al., 2004). The major effector of the RNA splicing reaction is the spliceosome, a complex of hundreds of interacting proteins, and small nuclear RNAs (snRNAs) including the small nuclear ribonucleoproteins (snRNPs) U1, U2, U4, U5, and U6 (Tazi et al., 2009). Each intron of the pre-mRNA is flanked by a 5'-exon and a 3'-exon and contains different conserved splicing signals recognized by the spliceosome: the 5'-splice site, the branch point sequence, the 3'-splice site, and the polypyrimidine tract located 5-40 bp upstream of the 3' end of the intron (Cartegni et al., 2002) (**Supplementary Figure 1**). Since these splicing signals are not sufficient for splicing regulation, the fidelity of pre-mRNA splicing depends on interactions between *trans*-acting factors (proteins and ribonucleoproteins) and *cis*-acting elements (pre-mRNA sequences), including exonic splicing enhancer (ESE), exonic splicing silencer (ESS), intronic splicing enhancer (ISE), and intronic splicing silencer (ISS) elements (Blencowe, 2006), that exert their effects by facilitating the binding of splicing factors, which in turn positively or negatively regulate inclusion of a particular exon.

Due to its underlying complexity, AS can lead to disease in different ways. The most common alterations of the splicing process are in *cis*-acting regulatory elements that are located either in core consensus sequences (5' splice site, 3' splice site, and branch point) or in regulatory elements that modulate spliceosome recruitment (Singh and Cooper, 2012). Some authors estimate that up to 62% of all disease-causing single nucleotide variants (SNVs) may affect RNA splicing (Lopez-Bigas et al., 2005). In terms of evolutionary conservation, about 50% of the synonymous positions in codons of conserved alternatively spliced mRNAs are under selection pressure, suggesting that conserved alternative exons and their flanking introns are strongly enriched in splicing regulatory elements (Blencowe, 2006). In this regard, it has been estimated that up to 25% of synonymous substitutions can disrupt normal splicing in the same way as non-synonymous variants or premature termination codons (Pagani et al., 2005), suggesting that those regions should also be routinely examined. Different examples of Mendelian disorders have already been associated with transcriptional perturbations introduced by both synonymous and non-synonymous variants (Slaugenhaupt et al., 2001; Cassini et al., 2019) (Supplementary Table 1). Since RNA-seq is not a part of current diagnostic genetic testing routine, these estimates seem to reflect a significant proportion of potentially diagnosable cases that remain unresolved at present. Some authors demonstrate the utility of RNA-seq to diagnose 10% of patients with mitochondrial diseases and identify candidate genes for the remaining 90% (Kremer et al., 2017).

## Section 1: Towards Clinical Application of RNA Sequencing

During the past years, the importance of RNA-seq as a clinical diagnostic tool has increased. The possibility to analyze new types of potential pathological variants in clinical routine has led to an increase in the diagnostic rate without an excessive increment in cost or time. However, some issues of RNA-seq analysis must be resolved to ensure the diagnostic quality of the study.

RNA-seq can complement the limitations of purely genetic information by probing variations in RNA with different additional studies (Kremer et al., 2017). First, the expression level of a gene or transcript outside of its physiological range can be measured. Second, cases with allele-specific expression (ASE), and therefore their association with disease predisposition, can be identified (Byron et al., 2016). Third, aberrant splicing can be recognized, which is known to be a major cause of Mendelian disorders (Tazi et al., 2009; Singh and Cooper, 2012; Scotti and Swanson, 2016).

Different studies suggest that 9 to 30% (Stenson et al., 2017) of disease-causing variants have an impact on RNA expression. The measurement of gene expression is thus expected to represent an improvement of the clinical routine; for example, some authors correlate the under-expression of certain genes with loss of function (LOF). This strategy has already been used in the identification of under-expression of *RARS2* in blood, which is associated with global developmental delay, seizures, microcephaly, hypotonia, and progressive scoliosis (Fresard et al., 2018).

Variable expressivity and incomplete penetrance are recurrent genetic issues in variant interpretation and may result from a combination of allelic variation, modifier genes, and/or environmental factors. A genetic condition with a reduced penetrance or high variability of symptoms may be a challenge for diagnosis. Allele-specific expression refers to the differential abundance of the allele copies and is thought to be relevant for as much as 50% of all human genes (Cooper et al., 2013). This differential allele expression can favor either the mutant or the wild-type allele and hence may influence clinical penetrance in different directions (Cartegni et al., 2002). Assuming a recessive condition, ASE-based analysis can help to reveal mono-allelic expression (MAE). For example, variants located in conserved splice sites of exon 12 of the *SPAST* gene lead to exon skipping and cause hereditary spastic paraplegia (HSP). Degradation of aberrant transcripts by a nonsense-mediated decay (NMD) mechanism results in ASE of the *SPAST* wild-type allele (Lopez-Bigas et al., 2005). In contrast, asymptomatic carriers of autosomal dominant retinitis pigmentosa (adRP) are protected from the disease by ASE of the wild-type *PRPF31* allele (Byron et al., 2016). In this context, ASE-based analyses may complement DNA resequencing studies such as WES or WGS for the identification of causative and low-frequency regulatory variants (Lappalainen et al., 2013) or disease-associated predisposition variants (Valle et al., 2008; De La Chapelle, 2009).

## Section 2: RNA-seq, Bioinformatics Approach and New Perspectives for Knowledge of Genetic Variation

RNA-seq data processing after NGS sequencing is mandatory for an appropriate analysis. As noted in Conesa et al. (2016)there is no optimal pipeline for all the different applications and scenarios in RNA-seq. However, data processing steps must be included in clinical routine in order to guarantee the quality and reproducibility of the study.

Usually RNA-seq data analysis must start with raw-data quality control. This allows obtaining a general idea of the quality of the sequencing and deciding if the quality requirements for the clinical routine are met. For this purpose different bioinformatics tools such as FastQC (Andrews, 2010) allow to control the most important and general parameters for global evaluation, such as Phred quality score, read length distribution, GC content, k-mer over-representation, adapter content, and duplicated reads. In case of adapter removal, specific bioinformatics tools may be necessary; some of the most referenced tools are CutAdapt (Compeau et al., 2013), FASTX-Toolkit (Gordon, 2010), and Trimmomatic (Bolger et al., 2014). For example, adapter presence or reduced read quality could lead to read misalignment or altered gene expression estimation and splicing event detection.

**Figure 1:**
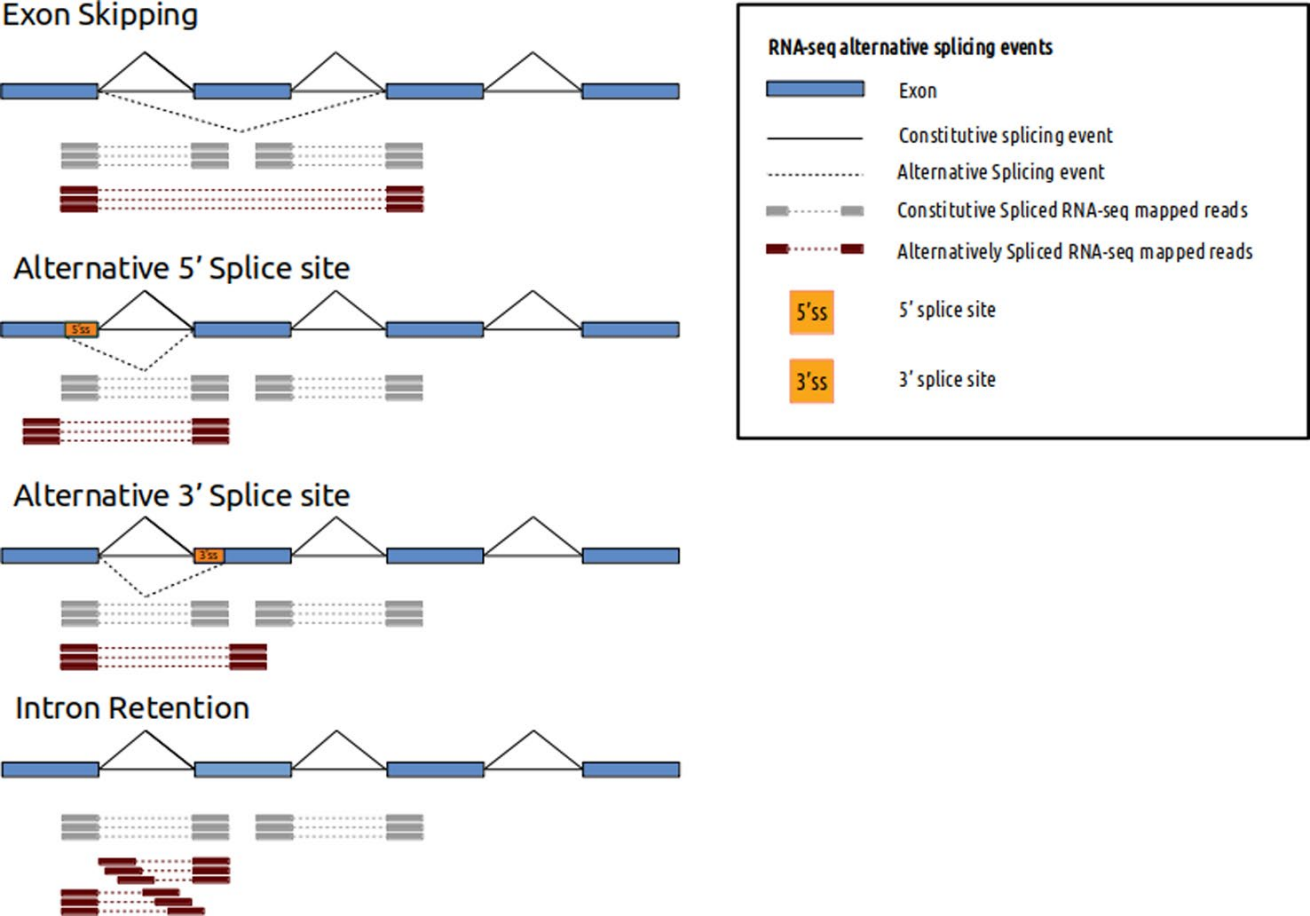
RNA-seq alternative splicing events and mapped reads. Different alternative splicing events can be detected using RNA-seq. Spliced mapped reads anchor differently if alternative splicing event occurs. Constitutive spliced RNA-seq mapped reads are represented in gray and alternatively spliced RNA-seq mapped reads are represented in red.

In the next step, raw-data reads are mapped against a human reference genome using a splice-aware alignment algorithm, such as STAR (Dobin et al., 2013), TopHat2 (Kim, 2013) or HiSAT2 (Kim et al., 2015). Splice-aware aligners allow reads to partially align into splice junctions between exons (**Figure 1**). In this step, there are important variables that must be evaluated and adjusted according to the type of study and phenotype. For example, the reference version of the genome (Guo et al., 2017) has an impact on the sensitivity and the specificity of variants identified. On the other hand, reference genome annotation files (such as bed or gtf) have a positive impact on mapping performance, quantification, and detection of differential expression and alternative splicing (Wu et al., 2013). To enrich reference genome annotation, some helpful databases that can be incorporated are SpliceDisease (Finotello et al., 2014) and ASpedia (Wang et al., 2016). SpliceDisease links experimentally supported and manually curated splicing-mutation disease entries with genes and diseases. ASpedia provides genomic annotations extracted from DNA, RNA and proteins, transcription, and regulatory elements obtained from NGS datasets, and isoform-specific functions collected from published datasets.

After mapping the reads to the genome, there are some technical and biological biases that can affect the sensitivity threshold. The 3' end bias of the mapped transcripts could either indicate a technical issue of reduced performance of the number of priming positions from which reverse transcriptase can start cDNA synthesis (Finotello et al., 2014) or a biological issue of RNA degradation by 5' exonuclease (Wang et al., 2016). Assessment of this type of bias is mandatory for the acceptance or rejection of clinical routine samples, and this can be done with quality control tools such as RSeQC (Li et al., 2015).

Prior to assessing differential expression of genes and their isoforms, mapped reads must be quantified. Tools like HTSeq (Anders et al., 2015), FeatureCounts (Liao et al., 2014), and GenomicAlignments (Lawrence et al., 2013) allow quantification of the number of mapped reads within a specific gene feature. Several biases like gene length (Gao et al., 2011) or GC content (Risso et al., 2011) may affect the quantification process and have a negative impact on the differential expression analysis (DEA). To reduce these biases, several methods have been described. Some methods normalize the read counts based on gene length and library size (total number of reads per replicate). As described in Conesa et al. (2016) the most employed methods involve the use of RPKM units (reads per kilobase of transcript and per million mapped reads) for single-end reads (Mortazavi et al., 2008), FPKM units for paired-end reads (fragments per kilobase of transcript per million mapped reads), and TPM units (transcripts per million). Other more complex normalization strategies are based on a theoretical initial distribution or on housekeeping genes (Evans et al., 2018).

At the isoform level, other quantification methods such as Cufflinks (Evans et al., 2018) and RSEM (Li and Dewey, 2011) are employed. Before testing differential expression between patients, it is mandatory to control technical batch effects and possible biological bias related to biopsy site, gender, or age. Principal component analysis (PCA) or Multi-Dimensional Scaling (MDS) are useful tools for monitoring these effects. After obtaining counts for the gene or transcript level, the count data is processed with different statistical methods such as R/Bioconductor package DESeq2 (Love et al., 2014), (Anders and Huber, 2010), edgeR (Robinson et al., 2009), or SVA (Leek et al., 2012). These tools use batch effect adjustment or modeling to reduce this technical bias. A whole functional RNA-seq pipeline provided by ENCODE can be found in: https://github.com/ENCODE-DCC/rna-seq-pipeline

Allele-specific expression can be identified by correlating allele counts obtained from RNA-seq and DNA resequencing. This comparison can be processed using pileLettersAt from the R/Bioconductor package GenomicAlignments (Lawrence et al., 2013). Some authors indicate that the sensitivity of ASE estimation depends on different technical variables such as variant coverage, allele frequency, or the number of alternative alleles (Kremer et al., 2017).

As stated in the American College of Medical Genetics guidelines (Richards et al., 2015), splice site prediction tools such as GeneSplicer (Pertea et al., 2001), Human Splicing Finder (Desmet et al., 2009), and MaxEntScan (Yeo and Burge, 2004) have a higher sensitivity (∼90–100%) relative to the specificity (∼60–80%) in predicting site abnormalities. It is recommended to use different algorithms to build a single piece of evidence regarding splice site variations. Other algorithms like LeafCutter (Li et al., 2018) rely on RNA-seq data and are able to identify variable splicing events such as: exon skipping, exon truncation, exon elongation, new exon, and complex splicing (or any other splicing event or combinations of the ones mentioned) using short-read RNA-seq data and focusing on excised introns (not relying on predefined models like other tools such as Cufflinks (Roberts et al., 2011)).

## Section 3: Issues to Be Addressed in the Transcriptomic Approach

Due to the dynamic nature of the transcriptome, RNA-seq studies present an important technical complexity. Even if RNA-seq studies can be introduced into clinical routine, some conceptual problems should be solved in the coming years.

Different authors point out that one of the major difficulties in transcriptomic analysis and its application to clinical routine is tissue-specific expression (Cummings et al., 2017), where genes and especially their isoforms can present a wide spectrum of splicing events and expression patterns depending on the tissue or cell type. This point is essential for a correct clinical interpretation of the variants (Melé et al., 2015), (Wang et al., 2008), but presents a problem in the initial selection of material for clinical routine. It is mandatory to assess invasiveness when obtaining the material related to the studied disease. Regarding this issue, it is documented that "noninvasible" material such as fibroblasts and blood present 68 and 70.6% of detectable expression of OMIM genes (Cummings et al., 2017; Fresard et al., 2018). This data indicates that using these tissues could help solve a broad spectrum of clinical studies using RNA-seq technology. For example, in neurological diseases, blood tissue presents a detectable expression of 76% of the genes associated with their phenotypes (Fresard et al., 2018).

However, tissue-specific expression may confound RNA-seq analyses and manifests the necessity to select the optimal tissue, whose basal gene expression profile allows monitoring all genes associated with the studied phenotype. For the efficient inclusion of RNA-seq analysis into clinical routine, new biological knowledge is required and additional bioinformatics tools need to be developed. In this context, new databases based on large-scale studies have been collecting and integrating information focused on the relationships between genes, isoforms, and tissues. The database established by the GTEX consortium is one of the most important and widely referenced databases (Melé et al., 2015). As noted in Cummings et al. (2017), the GTEX database is used for tissue selection depending on the clinical case. This information can become the mainstay of new algorithms for the *in silico* selection of optimal tissue depending on the specific disease or phenotype studied for clinical RNA-seq analysis. Some tools using such algorithms have already been described, such as for example PAGE ().

Additionally, this type of database homogenizes the transcriptomic information from large-scale analyses and could be a valuable source of control samples for statistical contrast and the identification of relatively high frequency variants or splicing events. For this initiative to succeed, and to overcome the inter-analysis barriers, the homogenization of sequencing protocols, starting materials, coverage of analysis, patient description, and bioinformatics pipelines is essential (Cummings et al., 2017). In addition, it is necessary to define the laboratory and bioinformatics parameters and tools that allow monitoring and controlling this process. For example, from a laboratory point of view, assessment of the quality and quantity of extracted RNA, or the library preparation strategy and its possible relationship with technical bias for the NGS process are some of the most important parameters to consider (Wai et al., 2019). To control this bias, different mathematical methods, such as principal component analysis (PCA) or t-Distributed Stochastic Neighbor Embedding (tSNE) based on expression have been proposed (Dey et al., 2017). Another important consideration is the definition of RNA spike-in control mixtures (Devonshire et al., 2010). These elements allow the evaluation of the technical and biological variability, and are essential for the identification of confounding effects, normalization processes, and quality control.

Regarding technical sensitivity and specificity of RNA-seq applied to clinical routine, the dynamic nature of transcriptomics and the complexity of some alterations, for example, splicing events or ASE deviation, multiplies the number of technical and biological variables to be considered during bioinformatics analysis (Costa et al., 2014). This complexity is reflected in the need to design mathematical methods capable of absorbing if not all, at least part of the variability present in this type of study. In this respect, there are different obstacles for bioinformatics analysis of RNA-seq data. Among them are the mapping process and the possible effect of different factors on the identification of variants, such as the presence of neighboring SNPs and small indels in the unbiased identification of ASE (Wood et al., 2015; Byron et al., 2016), junction events (Williams et al., 2014), or the isoform assembly process, where the length of reads, library preparation strategy, the initial coverage, and GC content of the transcripts could affect the accuracy of the transcript identification process (Mantere et al., 2019;Wai et al., 2019).

## Final Remarks

The RNA-seq approach holds the promise to become an interesting clinical routine tool to increase the genetic diagnostic rate. This methodology may increase our knowledge about genetic alterations and their association to genetic diseases with the inclusion of other types of variants, such as splicing events or aberrant gene expression. This type of alterations is usually not detected by DNA resequencing analyses and may be one of the main reasons of the moderate diagnostic rate of this methodology in some diseases.

However, due to the dynamic nature of the transcriptome, RNA-seq analysis presents a high complexity, with the concomitant need to consider different technical and biological variables. The control and the effect of these possible fluctuations are currently under investigation. In this context, a deeper and more specific knowledge of the technical and bioinformatics area that varies with the analyzed disease seems necessary to guarantee a meaningful clinical outcome. In this sense, great advances are being made in bioinformatics to define, homogenize, and monitor the transcriptomic information in order to break the inter-analysis barrier, which is mandatory for clinical reproducibility. However certain issues remain outstanding that should be further defined and resolved in the coming years.

## Author Contributions

All authors contributed to manuscript writing, revision, read and approved the submitted version.

## Funding

JB’s lab is partially funded by grant PI16/00440 from Instituto de Salud Carlos III (ISCIII), cofunded by European Regional Development Fund (ERDF).

## Conflict of Interest

The authors declare that the research was conducted in the absence of any commercial or financial relationships that could be construed as a potential conflict of interest.
