# Supplementary material for: RNA-Seq Perspectives to Improve Clinical Diagnosis
Source: Front Genet. 2019 Nov 12;10:1152. doi: 10.3389/fgene.2019.01152 (PMC6861419; doi:10.3389/fgene.2019.01152)
Supplement: Supplementary file 1 [file DataSheet_1.pdf]

frontiers

Supplementary Material

1 Supplementary Figures and Tables

1.1 Supplementary Figures

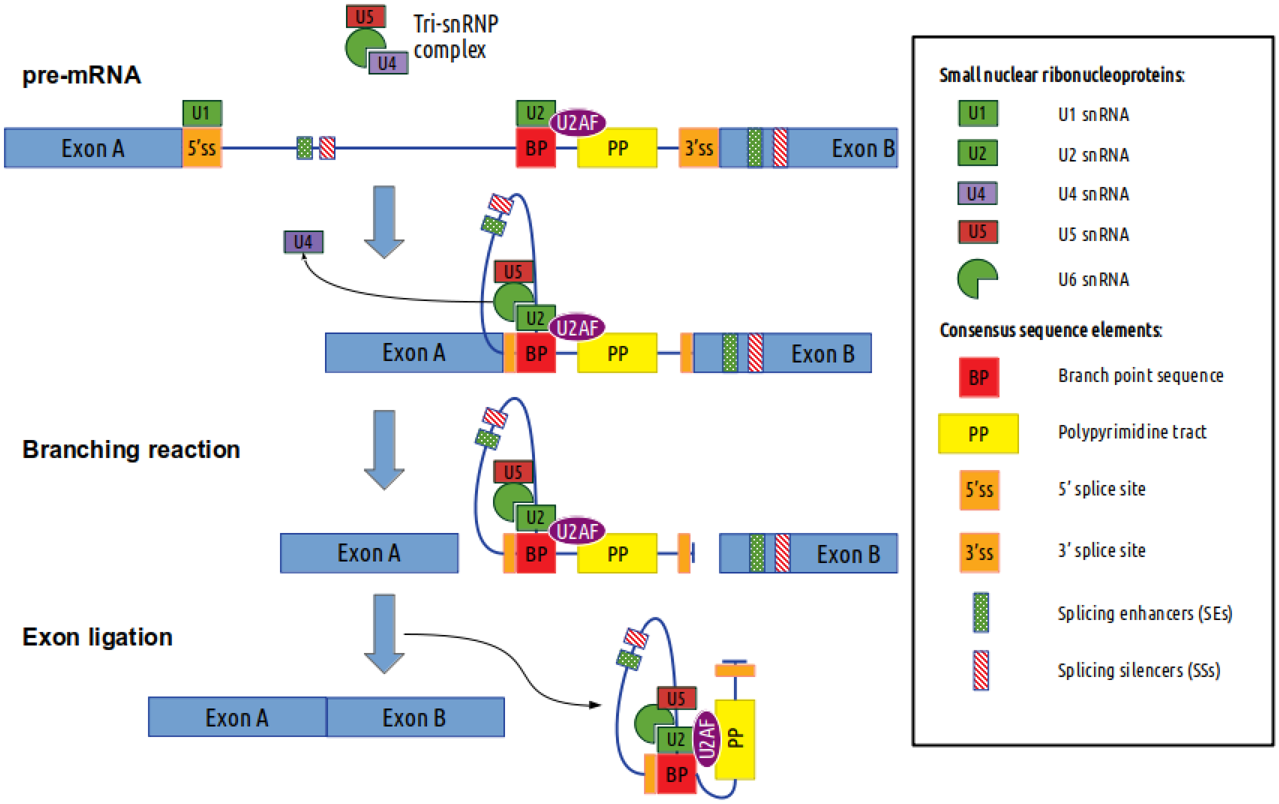

Supplementary Figure 1. RNA splicing process. Splicing can be divided into two main steps: the branching reaction that generates a free the 5'-exon and an intron lariat-3'-exon intermediate; and exon ligation that joins the 5'-exon and the 3'-exon while removing the intron lariat. The assembly of the spliceosome begins with the recognition and binding of the 5'-splice site by U1 small nuclear ribonucleoprotein (snRNP), the branch point sequence by U2 snRNP, and the polypyrimidine tract by U2 auxiliary factor (U2AF), which facilitates correct U2 binding. U2 recruits a tri-snRNP complex containing U4, U5 and U6 to form the fully assembled complex. Activation of the spliceosome requires the dissociation of U4 snRNP, allowing U6 to replace U1 at the 5'-splice site and leading to a U6-U2 interaction that brings the 5'-splice site and the branch point close together. Once activated, the branching reaction and exon ligation occur, U5 brings the two exons into close proximity and allows exon ligation.

1.2 Supplementary Tables

Supplementary Table 1. Different examples of Mendelian disorders associated with transcriptional perturbations

Disease	Gene	Mutation	Molecular mechanism	Splicing effect	Reference
**cis-regulatory elements**					
Spinal muscular atrophy (SMA) and medium-chain acyl CoA dehydrogenase deficiency (MCAD)	SMN2	C > T transition at position +6	Creates an ESS allowing a binding site for hnRNPA1	Exon 7 skipping	(Kashima et al. 2007)
Frontotemporal dementia with parkinsonism linked to chromosome 17 (FTDP-17)	MAPT	c.892A>G	Abrogates an ESS	Exon 10 inclusion	(Iovino et al. 2014)
Hutchinson-Gilford progeria syndrome (HGPS)	LMNA	c.1824C>T	Alternative 5’ splice site	150 nt deletion in exon 11	(Eriksson et al. 2003)
Familial dysautonomia (FD)	IKBKAP	c.2204+6 T>C	Disrupts binding of U1 to the 5'-splice site	Exon 20 skipping	(Cheishvili et al. 2007)
Menkes disease (MNK)	ATP7A	Substitutions at IVS6	Alternative donor splice site	Exon skipping of exon 6 or exon 6 and 7	(Møller et al. 2002)
Menkes disease (MNK)	ATP7A	Substitutions at IVS6	Activation of a cryptic donor splice site	Exon 6 enlarged with 50bp, leading to frameshift	(Møller et al. 2002)
Myotonic dystrophy type I (DM1)	DMPK	CTG expansion in the 3’ UTR	Repeat containing transcripts accumulate in nuclei and alter the function of RNA binding protein	Alternative splice deregulation	(Kuyumcu-Martinez, Wang, and Cooper 2007)
Prostate cancer progression	KLF6	IVS1 – 27 G>A/IVSΔA	Novel functional SRp40 DNA binding site, ablating two other overlapping SR-protein binding sites	New isoforms that act as a dominant negative	(Narla et al. 2005)
GISTs	KIT	25-bp deletion of exon 11	Novel 3'-splice acceptor site	Constitutive activation of a mis-spliced KIT isoform	(Chen et al. 2005)
Spinal muscular atrophy (SMA)	SMN1	c.922+6 G>T	Disrupts the 5' splice donor site	Exon skipping	(Lorson et al. 1999)
Breast cancer	BRCA1	G > T substitution at nucleotide 5199	Abrogates a putative ESE	Exon 18 skipping	(Mazoyer et al. 2002)
Ehlers-Danlos syndrome (EDS)	COL5A1	IVS6-2A>G	Disrupts the acceptor splice site	Exon 7 skipping	(Symoens et al. 2011)
Congenital cataract	MIP	c.606+1G>A (IVS3)	Novel splice site	Exon 3 skipping	(Zeng et al. 2013)
SCOT deficiency	OXCT1	c.1248+5G>A (IVS13)	Disrupts the donor splice site	Exon 12 and 13 skipping	(Hori et al. 2013)
Neurofibromatosis type 1 (NF1)	NF1	G-to-A transition at position +1 of the 5’ splice site of exon 12a	Abrogates the 5’ splice site of exon 12a	Exon 11 and 12a skipping	(Fang et al. 2001)
Duchenne muscular dystrophy (DMD)	DMD	Intron 45+1G>A	Activation of cryptic donor splice site	Partial loss of exon 45	(Habara et al. 2009)
**trans-acting protein factors**					
Dilated cardiomyopathy (DCM)	RBM20	c.1962T>G	Alteration of RNA binding domain R/S	Aberrant splicing of TTN gene	(Guo et al. 2013)
**Spliceosome assembly**					
Autosomal dominant retinitis pigmentosa (adRP)	PRPF6	c.2185C>T	Altered nuclear localization	Affected U4/U6 interaction leading to alteration of spliceosome assembly	(Tanackovic et al. 2011)
